# Aromatic pollutants rewire soil microbial carbon fixation via chain elongation

**DOI:** 10.1093/ismejo/wraf254

**Published:** 2025-11-25

**Authors:** Qing-Lian Wu, Tian Lan, Lin Deng, Jing-Wen Jia, Wei-Tong Ren, Hua-Zhe Wang, Juan-Shan Du, Nan-Qi Ren, Wan-Qian Guo

**Affiliations:** State Key Laboratory of Urban-rural Water Resource and Environment, Harbin Institute of Technology, Harbin 150090, China; State Key Laboratory of Urban-rural Water Resource and Environment, Harbin Institute of Technology, Harbin 150090, China; State Key Laboratory of Urban-rural Water Resource and Environment, Harbin Institute of Technology, Harbin 150090, China; State Key Laboratory of Urban-rural Water Resource and Environment, Harbin Institute of Technology, Harbin 150090, China; State Key Laboratory of Urban-rural Water Resource and Environment, Harbin Institute of Technology, Harbin 150090, China; State Key Laboratory of Urban-rural Water Resource and Environment, Harbin Institute of Technology, Harbin 150090, China; State Key Laboratory of Urban-rural Water Resource and Environment, Harbin Institute of Technology, Harbin 150090, China; State Key Laboratory of Urban-rural Water Resource and Environment, Harbin Institute of Technology, Harbin 150090, China; State Key Laboratory of Urban-rural Water Resource and Environment, Harbin Institute of Technology, Harbin 150090, China

**Keywords:** BTEX pollution, chain elongation, medium-chain fatty acids, soil, carbon sequestration, metagenomic, metaproteomic

## Abstract

Widespread aromatic pollutants such as benzene, toluene, ethylbenzene, and xylene are traditionally considered to drive soil carbon loss through mineralization and ecotoxicity. Contrary to this view, our study reveals that low concentrations of these pollutants stimulate microbial carbon chain elongation—a previously overlooked carbon conversion pathway producing medium-chain fatty acids, thereby reshaping soil carbon dynamics. Using phased amplicon sequencing, metagenomics, and metaproteomics of soil microcosms amended with these compounds, we demonstrate that aromatic pollutants bidirectionally regulate carbon chain elongation at both taxonomic and molecular levels. These pollutants selectively enrich *Clostridium sensu stricto 12* and *Rummelibacillus* while suppressing *Acinetobacter*, a key elongation taxon in natural soils. Simultaneously, they inhibit *Petrimonas*, a syntrophic fatty acid degrader, enhancing medium-chain fatty acids accumulation. Carbon chain elongating bacteria cooperate with aromatic degraders, redirecting pollutant-derived carbon towards chain elongation rather than complete mineralization to CO₂. Among them, *Bacillus* occupies a pivotal niche bridging aromatic degradation and carbon elongation. At the molecular level, aromatic pollutants enhance chain elongation by accelerating substrate uptake and channeling acetyl-CoA into the reverse β-oxidation pathway. Additionally, aromatic pollutants inhibit the fatty acid biosynthesis pathway by upregulating *fabR* and down-regulating *acrR* and *fadR*. They also maintain NADH availability to alleviate Rex-mediated repression of *bcd*, a critical gene in the β-oxidation pathway. However, high concentrations of aromatic pollutants disrupt metabolic homeostasis and suppress chain elongation activity. Our findings redefine the ecological impact of aromatic hydrocarbon contamination in soil, demonstrating their role in modulating anaerobic carbon fixation and retention within soil microbial communities.

## Graphical Abstract

**Graphical Abstract:**
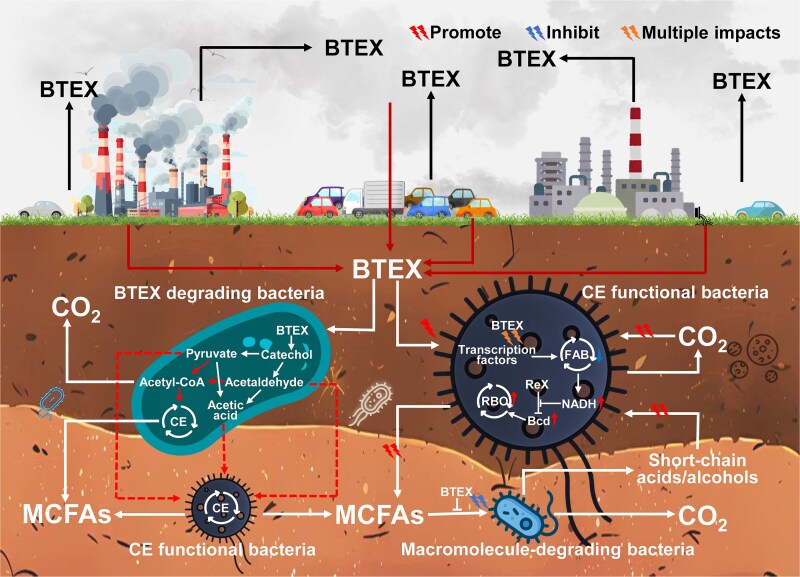

## Introduction

Soil represents the largest terrestrial carbon reservoir and a pivotal nexus in global biogeochemical cycles, where the microbiome acts as a fundamental driver of carbon transformation and turnover [1]. In recent decades, microbially mediated carbon chain elongation (CE) has been identified as a significant microbial carbon transformation process, through which short-chain carboxylic acids and alcohols can be converted into more stable medium-chain fatty acids (MCFAs) [2–4]. CE-capable microbial consortia are widely distributed across soil ecosystems [5, 6], with mounting evidence establishing CE as a fundamental microbial carbon metabolism pathway in soils [7, 8]. This process transforms small-molecular substrates in the soil into MCFAs that exhibit high resistance to microbial degradation, thereby contributing to aromatic carbon stabilization within the soil matrix and mitigating carbon loss via CO₂ or CH₄ emissions [9]. However, the ecological drivers governing CE, its underlying regulatory mechanisms, and resilience to environmental disturbances remain poorly understood.

Anthropogenic aromatic pollutants constitute a particularly important yet underexplored class of potential disruptors to microbial carbon transformations. Among soilborne anthropogenic stressors, benzene, toluene, ethylbenzene, and xylene (BTEX) are pervasive aromatic pollutants arising from fossil fuel combustion, petrochemical spills, and industrial discharge [10–12]. Most existing studies have centered on the aerobic and anaerobic microbial degradation pathways of BTEX, or its cometabolism with other aromatic pollutants such as halogenated hydrocarbons [13, 14], as well as its toxicological impacts on the human cardiovascular and respiratory systems [15, 16]. In contrast, their broader ecological implications, particularly their impact on microbial carbon metabolism in soil, have received limited attention. Microbial mineralization of BTEX compounds can lead to substantial CO₂ and CH₄ emissions, constituting a well-documented pathway of carbon loss from soil systems [17, 18]. Additionally, BTEX may impair microbial physiological functions and disrupt key ecological processes, potentially altering soil carbon storage capacity [19]. However, an overlooked aspect is that intermediate metabolites of BTEX degradation, such as pyruvate, acetaldehyde, and acetyl-CoA [20], could serve as critical substrates fueling CE, potentially mitigating carbon loss by redirecting carbon flux toward more stable forms. Recent reports that certain phenolic compounds [21] and nanopollutants [22] can promote CE raise the question of whether BTEX compounds might similarly stimulate CE, thereby enhancing soil carbon retention.

Given the growing global interest in using microbial processes for carbon sequestration and soil rehabilitation [23, 24], resolving how aromatic pollutants modulate carbon sequestration pathways is both scientifically and environmentally urgent. Deciphering the regulatory mechanisms through which CE responds to BTEX perturbation will advance our knowledge of the fate of aromatic hydrocarbons in soils and their potential contributions to microbial carbon stabilization.

To address these questions, we used a time-resolved multi-omics strategy combining phased amplicon sequencing, metagenomics, and metaproteomics to investigate BTEX concentration–dependent effects on CE activity in soil microcosms. By linking microbial community succession, interspecies interactions, and molecular-level functional dynamics, we aim to uncover how BTEX influences carbon allocation patterns and regulatory networks in soil microbiomes. This work provides critical insights into how aromatic pollutants (e.g. BTEX) modulate microbial carbon transformation and highlights CE as a potentially resilient pathway for carbon stabilization in contaminated soils.

## Materials and methods

### Soil sampling and analysis

In September 2024, we collected soil samples for the first time at a natural wetland in Heilongjiang Province, China (45°45′13″N, 126°32′24″E), a remote area with minimal impact from human activities. A five-point composite sampling strategy was employed, with ~10 m spacing between points. After removing surface litter, soils from 0–20 cm depth were obtained using presterilized stainless-steel corers. Samples were transferred into sterile polyethylene bags, stored on dry ice in light-shielded containers, and transported to the laboratory within 4 h. In a laminar-flow hood, soils from each sampling point were gently passed through a sterile 2 mm sieve to remove stones and plant debris, and subsequently homogenized by manual mixing in sterile trays until a uniform texture was achieved. Homogenized soils were stored at 4°C in the dark, and microcosm inoculations were initiated within 24 h. In September 2025, we collected additional soil samples from nine sites across three wetlands in Heilongjiang Province, all located away from urban areas, and subsequently reran the corresponding microcosm experiments to evaluate the generalizability of the observed microbial responses. Sampling locations are depicted in Fig. S1. BTEX compounds were below the detection limits in all pristine soils (benzene, 3.6 μg/kg; toluene, 3.2 μg/kg; ethylbenzene, 2.8 μg/kg; *p*- and *m*-xylene, 3.5 μg/kg; and *o*-xylene, 2.4 μg/kg).

### Microcosm construction

Pollutant levels were defined according to the “Soil environmental quality risk control standard for soil contamination of development land” (GB36600-2018) issued by the Ministry of Ecology and Environment of China. Three contamination regimes were established: slight (AS), below the class I screening threshold; moderate (AM), approximating the midpoint between the class I control limit and the class II screening value (±10%); and high (AH), exceeding the class II control limit. In addition, a noncontaminated group (A) was set up as a control group. Specific BTEX concentrations are listed in Table S1. Microcosms were assembled in 500 ml sterile fermentation vessels containing 150 g of homogenized soil and 300 ml of autoclaved medium (composition detailed in Table S2), which served as both substrate and nutrient source. Acetic acid (60 mM) and ethanol (120 mM) were supplied as sole carbon inputs, and BTEX compounds were added at predefined concentrations to simulate contaminant stress. To mimic natural conditions, headspaces were not flushed with nitrogen. Incubations were conducted at 30°C with constant agitation (160 rpm). The pH was maintained at 6.5 ± 0.1 via adjustment with sterile 6 M NaOH or HCl every 4 days. All the treatments were performed in triplicate. A complete overview of the experimental design is provided in Table S3.

### Chemical analytical methods

Short-chain alcohol, short-chain fatty acids (SCFAs), and MCFAs were quantified using gas chromatography (GC). BTEX concentrations were determined via gas chromatography–mass spectrometry (GC–MS). MCFAs selectivity, electron transfer efficiency, and carbon flux were calculated following our previously established protocols [25]. Detailed analytical procedures are provided in Text S1. Acids and alcohols were quantified and reported in millimole per kilogram, given their direct involvement in microbial biochemical processes and metabolic fluxes. In contrast, BTEX compounds were measured in milligram per kilogram to better reflect their environmental concentrations and toxicological relevance. The conversion between millimole per kilogram and milligram per kilogram for all quantified compounds is provided in Table S4.

### Extraction and quantification of NAD^+^ and NADH

On Day 12, 0.5 g of soil slurry was collected from each microcosm and centrifuged at 5000 rpm for 5 min at 4°C. The supernatant was discarded, and the pellet was used for intracellular coenzyme extraction. NAD^+^ and NADH concentrations were determined using a commercial assay kit (NAD^+^/NADH Quantification Kit, Nanjing Jiancheng Bioengineering Institute, A114-1-1) following the manufacturer’s instructions.

### Amplicon sequencing and diversity analysis

Soil slurry samples from treatments A, AS, AM, and AH were collected on Days 0 (native soil), 8, 16, and 24 for DNA extraction and 16S rRNA genes amplicon sequencing. Detailed protocols for DNA extraction, PCR amplification, sequencing, and bioinformatic processing are provided in Text S2. Alpha and beta diversity analyses based on 16S rRNA gene data were performed to assess microbial community composition and structure across treatments and time points. Analytical methods and statistical approaches are described in Text S3.

### Metagenomic analysis

On the 24th day, DNA was extracted from samples of A, AS, AM, and AH, followed by metagenomic sequencing to profile functional potential. Full methodological details are available in Text S4.

### Metaproteomic analysis

On the 24th day, proteins were extracted from treatments A and AS and subjected to metaproteomic analysis to resolve functional expression patterns. The complete extraction and analysis protocols are described in Text S5.

### Data analyses

Statistical analyses were conducted using SPSS Statistics 27.0 (IBM Corp., Armonk, NY, USA). The Shapiro–Wilk test was applied to evaluate the normality of data distribution within each treatment group. For normally distributed data, homogeneity of variances was verified using Levene’s test, followed by independent-samples *t*-tests to assess pairwise differences between groups. For datasets that did not meet the normality assumption, nonparametric Mann–Whitney *U* tests were performed instead. A two-tailed *P* value < .05 was considered statistically significant.

## Results

### Effects of BTEX on CE in natural soil

**Figure 1:**
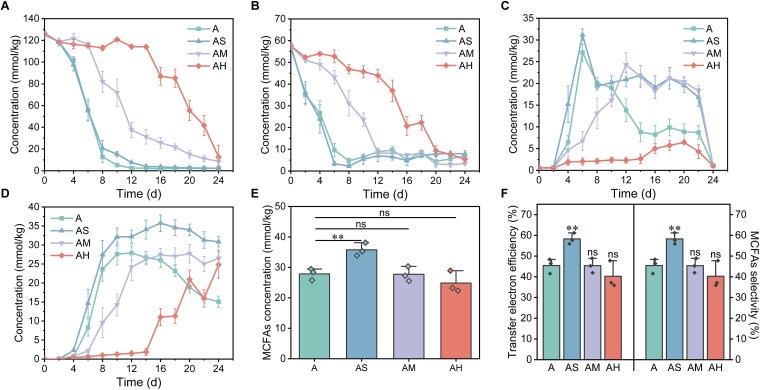
Contaminant, substrate and product concentrations, and associated correlation indicators. (A–D) Temporal concentration profiles of ethanol (A), acetic acid (B), butyric acid (C), and caproic acid (D) in treatments A, AS, AM, and AH over the course of the experiment. (E) Maximum accumulation of MCFAs, represented by caproic acid. (F) Electron transfer efficiency and MCFA selectivity corresponding to the maximum accumulation of caproic acid. ^**^indicates *P* < .01.

To assess the influence of BTEX stress on soil CE, we compared the caproic acid biosynthetic capacity in soils with varying levels of BTEX contamination. Under low contamination (Fig. 1A–D), substrates were rapidly consumed, and the synthesis and conversion of butyric acid (CE intermediate) were markedly accelerated. Caproic acid yield peaked in the AS (35.76 ± 2.17 mmol/kg), significantly exceeding that of other treatments. These findings suggest that low BTEX concentrations enhance CE activity by promoting faster and more efficient conversion of SCFAs and ethanol into MCFAs, thereby reinforcing carbon retention in soils. This stimulatory effect may reflect an enhanced microbial capacity to capture acetic acid and ethanol and redirect them into acetyl-CoA, the central CE precursor, facilitating more rapid substrate channeling into CE [26, 27]. Correspondingly, under AS conditions, the maximum accumulation of medium-chain fatty acids (MCFAs) (*t* = −4.746, df = 4, *P* = 0.009; Fig. 1E), selectivity (*t* = −4.996, df = 4, *P* = 0.008; Fig. 1F), and electron recovery efficiency (*t* = −4.996, df = 4, *P* = 0.008; Fig. 1F) were all significantly enhanced, with carbon fluxes were more strongly directed toward caproic acid. This redistribution favors the long-term stabilization of carbon in the form of MCFAs.

However, under moderate and heavy BTEX stress, the lag phase of caproic acid production showed dose-dependent prolongation. Although negligible effects on MCFA production performance were observed for AM, it led to a low decrease in MCFA yield and selectivity for AH (*t* = 1.311, d*f* = 4, *P* = .260). In addition, MCFA degradation was suppressed under all BTEX concentrations, highlighting a potential mechanism for enhanced carbon retention in contaminated soils (Fig. 1D). Because the synthesis and degradation of MCFAs are closely coupled to microbial community function, shifts in taxonomic and functional profiles likely contribute to the observed changes in carbon flux and stability [28].

Furthermore, to assess the generality of this phenomenon, we conducted parallel microcosm experiments using soils collected from nine sites across three remote wetlands. The experiments yielded reproducible results consistent with those observed in our primary study (Text S6).

### Succession of microbial communities under BTEX pollution

To elucidate the ecological impact of BTEX contamination on soil microbial communities, we analyzed community composition, diversity, and succession across four treatments. Rarefaction curves approached saturation (Fig. S2) and high Good’s coverage values (Table S5) confirmed sufficient sequencing depth, ensuring robust representation of microbial taxa. Alpha diversity analyses (Table S5) revealed a pronounced increase in richness and evenness under BTEX exposure. Compared with the uncontaminated control, all BTEX-amended groups exhibited elevated richness estimators (Sobs, Ace, Chao), as well as higher Shannon and phylogenetic diversity indices, demonstrating that BTEX contamination not only expanded microbial taxonomic breadth but also enhanced community evenness. Beta diversity analyses supported these findings. Hierarchical clustering (Fig. S3) and principal coordinates analysis (Fig. S4) (PCoA; ANOSIM, *r* = 0.57, *P* = .002) revealed distinct community separations between the control and BTEX-treated groups at each sampling stage, highlighting the strong selective pressure imposed by aromatic hydrocarbon exposure on soil microbial assembly.

**Figure 2:**
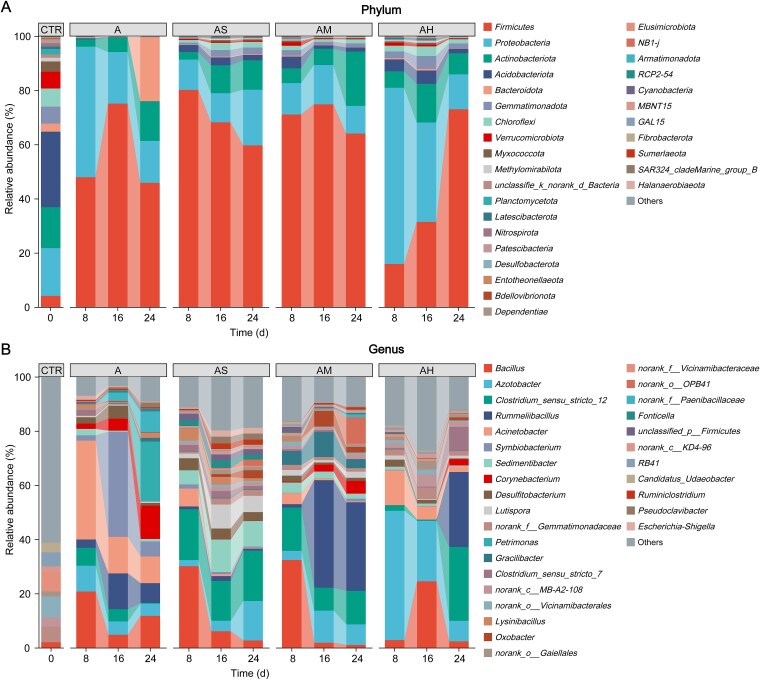
Temporal dynamics of microbial taxonomic composition under different BTEX treatments. Microbial communities are displayed at the (A) phylum and (B) genus levels. Data represent the mean relative abundance across sampling Days 0, 8, 16, and 24. Facet labels denote the treatment conditions: A (control), AS (soil with slight BTEX exposure), AM (soil with moderate BTEX exposure), and AH (soil with high BTEX exposure).

To assess the ecological consequences of BTEX-induced selective pressure, taxonomic variations at both the phylum (Text S7) and genus levels were analyzed throughout the incubation period. CE-associated genera exhibited pronounced BTEX-dependent shifts. Under BTEX exposure, *Clostridium sensu stricto 12* maintained dominance throughout the entire incubation period in AS and AM treatments, whereas *Rummeliibacillus* became dominant during the mid-to-late stages of AM and AH treatments, effectively replacing *Acinetobacter* (Fig. 2B), which was prevalent in the control group. In contrast, *Petrimonas* exhibited a clear temporal increase in the control group, transitioning from near absence in the early stage to high abundance at the end of incubation. Belonging to the phylum Bacteroidota, *Petrimonas* was mainly detected during the late phase when caproic acid concentrations decreased, coinciding with the reduction of accumulated MCFAs.

These results demonstrate distinct shifts in the composition and relative abundance of CE-associated genera under BTEX exposure, with the AS group showing characteristic enrichment of syntrophic and fermentative taxa compared with both the control and other BTEX levels.

### BTEX contamination rewires microbial metabolic networks to couple aromatic degradation with CE

**Figure 3:**
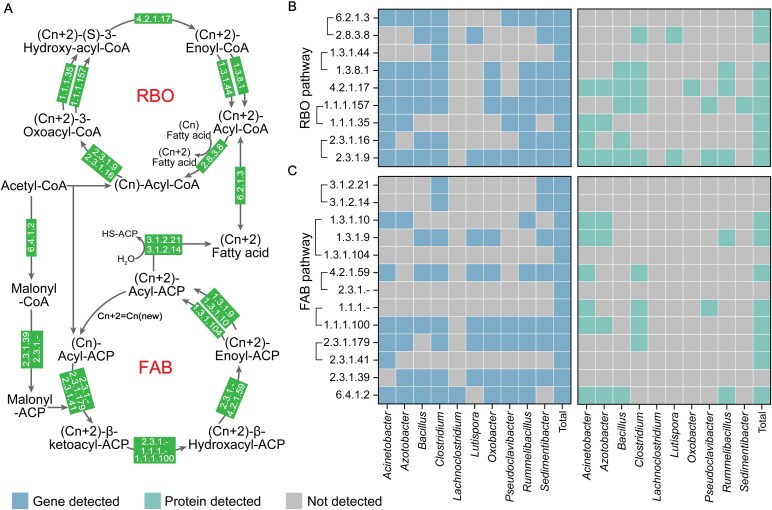
Classification and functional annotation of key genes and proteins involved in carbon elongation (CE) via the reverse β-oxidation (RBO) and fatty acid biosynthesis (FAB) pathways in the AS treatment. (A) Schematic representation of the RBO and FAB metabolic pathways. (B) Prevalence and distribution of RBO-related genes and their corresponding proteins across different bacterial genera. (C) Prevalence and distribution of FAB-related genes and their corresponding proteins across different bacterial genera. Gene abundances are expressed as Transcripts Per Million (TPM), and protein abundances as Label-Free Quantification (LFQ) intensities.

Microbial community composition and its temporal dynamics profoundly shape interspecies functional interactions. To assess how BTEX exposure modulates CE-associated metabolic cooperation among bacterial taxa, we conducted time-resolved correlation analyses between substrate/product concentrations and microbial abundances (Fig. S5). Compared to the uncontaminated control (A), BTEX-amended microcosms exhibited several genera (*Sedimentibacter*, *Lutispora*, *Oxobacter*, *Lachnoclostridium*, and *Pseudoclavibacter*) that revealed strong positive correlations with caproic acid accumulation, despite no prior evidence linking them to CE-related metabolism (Fig. S5). To evaluate the potential involvement of these taxa in CE–BTEX metabolic cross-talk, we reconstructed metabolic pathways at the gene and protein levels. These analyses included the aforementioned genera as well as the five most dominant genera in BTEX-treated microcosms (Fig. 2B). Integrated functional profiling (Fig. 3) revealed a complete reverse β-oxidation (RBO) pathway and a nearly complete fatty acid biosynthesis (FAB) pathway in the AS group, the latter lacking hydrolases corresponding to EC 3.1.2.21 or EC 3.1.2.14 (Fig. 3).

**Figure 4:**
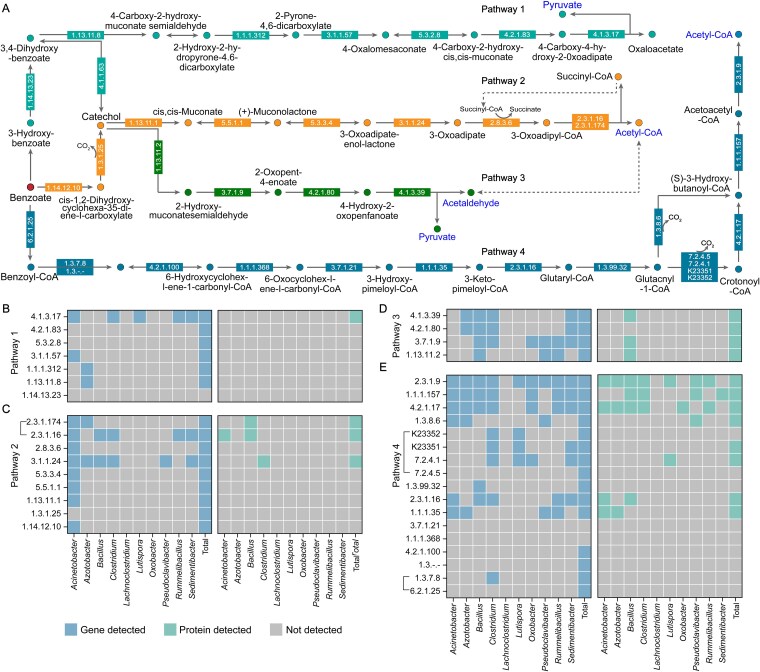
Distribution of benzoate and catechol degradation pathways and associated genes and proteins in the AS microcosm. (A) Schematic representation of the four major degradation pathways. (B–E) Heatmaps showing the genus-level distribution of pathway-specific genes and proteins across pathways 1–4. Enrichment of complete genes and proteins for pathway 3 indicates active utilization of this pathway for benzoate and catechol degradation. *Bacillus* exhibits complete pathway 3 genes and nearly complete protein coverage, highlighting its central functional role in mediating degradation. Gene abundances are expressed as TPM, and protein abundances as LFQ intensities.

During incubation, BTEX concentrations decreased significantly (Fig. S6A–C), suggesting active microbial transformation. Subsequent analyses of the key aromatic intermediates, benzoate and catechol, identified four potential downstream routes in the AS group (Fig. 4). Among them, pathway III was supported by both genomic and proteomic evidence, with catechol converted to acetaldehyde and pyruvate. These intermediates can permeate cell membranes and serve as precursors for acetyl-CoA formation, connecting aromatic compound degradation with CE-related carbon flux. *Bacillus* encoded and expressed nearly the full complement of pathway III genes and proteins (Fig. 4), highlighting its central role among BTEX-responsive taxa in bridging aromatic degradation and carbon elongation metabolism.

### BTEX-induced optimization of carbon assimilation toward acetyl-CoA

**Figure 5:**
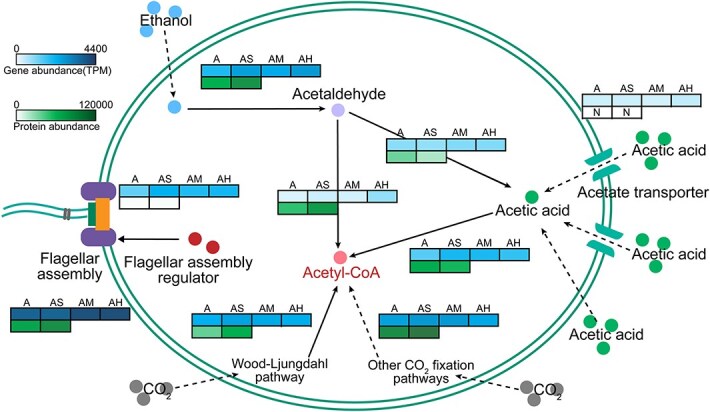
Multi-omics representation of substrate assimilation and acetyl-CoA biosynthesis within each microcosm. This stepwise schematic integrates metagenomic (gene-level) and metaproteomic (protein-level) data. Arrows denote enzymatic transformations along the pathway. The figure highlights the enhanced assimilation of exogenous and endogenous substrates by soil microorganisms under BTEX-enriched conditions and their directed flux toward acetyl-CoA biosynthesis. Gene abundances are expressed as TPM, and protein abundances as LFQ intensities.

To elucidate how BTEX exposure accelerates microbial carbon flux through CE, we examined microbial capacities for substrate acquisition and metabolic integration. Low and moderate BTEX concentrations exerted negligible effects on the abundance of acetate transporters, whereas high BTEX levels increased their relative abundance. Concurrently, BTEX exposure led to upregulation of genes and proteins associated with flagellar biosynthesis and motility regulation, reflecting enhanced cellular machinery related to substrate accessibility (Fig. 5).

In parallel, BTEX exposure modulated endogenous CO₂ fixation pathways in a concentration-dependent manner. Under low BTEX stress (AS), the expression of genes and proteins affiliated with the Wood–Ljungdahl (WL) pathway and other CO₂ assimilation routes increased, whereas this enhancement diminished at higher BTEX levels (Fig. 5). Such patterns indicate a graded regulatory response in microbial autotrophic capacity across BTEX concentrations.

Within ethanol catabolism, BTEX exposure altered carbon routing from ethanol oxidation to acetyl-CoA formation. Genes and proteins catalyzing the conversion of ethanol to acetyl-CoA were enriched, whereas those associated with the competing acetaldehyde-to-acetate branch were reduced (Fig. 5). In addition, ATP-dependent acetate reactivation enzymes showed lower abundance, consistent with a shift toward energy-efficient carbon utilization. Collectively, BTEX exposure induced coordinated changes in substrate uptake, intracellular carbon assimilation, and downstream acetyl-CoA allocation, reflecting concentration-dependent reorganization of microbial metabolism under chemical stress.

### BTEX-induced redistribution of acetyl-CoA flux

**Figure 6:**
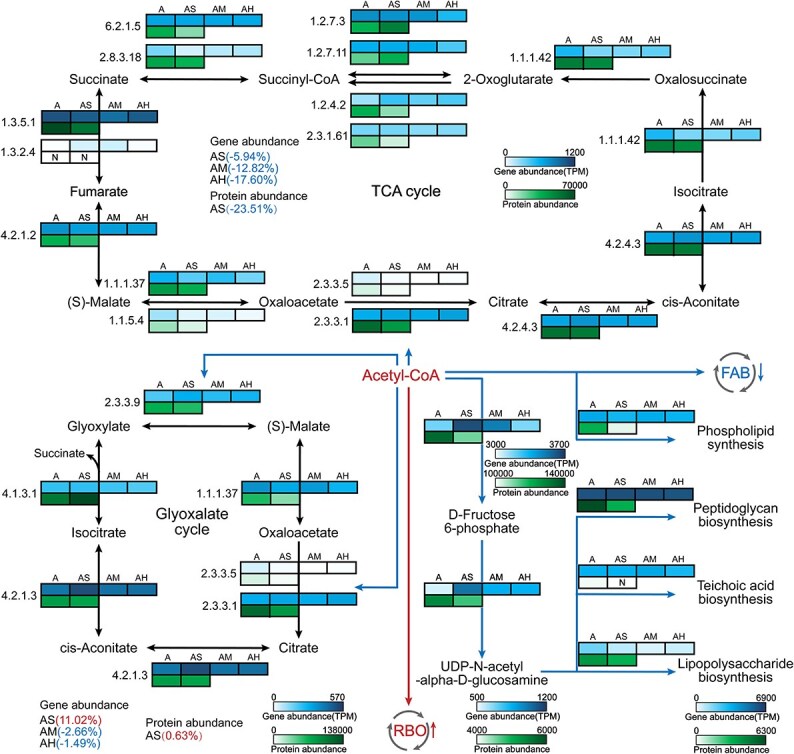
Metabolic remodeling of acetyl-CoA utilization in BTEX-exposed microcosms. This schematic illustrates the selective upregulation of the RBO pathway alongside suppression of central carbon metabolism. The figure highlights how BTEX exposure redirects carbon flux toward the RBO pathway. Gene abundances are expressed in TPM, and protein abundances in LFQ intensities.

The distribution of acetyl-CoA among competing metabolic routes is a critical determinant of CE efficiency and specificity. To examine metabolic remodeling under BTEX exposure, we analyzed key acetyl-CoA utilization pathways at the gene and protein levels. Despite a general repression of central carbon metabolism, the RBO pathway—the core route of CE—exhibited higher relative abundance, suggesting enhanced channeling of acetyl-CoA toward MCFAs biosynthesis (Fig. 6).

In the BTEX-treated AS group, proteins associated with the tricarboxylic acid (TCA) cycle were markedly downregulated. BTEX exposure also selectively reduced flux through the glyoxylate shunt by suppressing several key catalytic enzymes (EC 2.3.3.9, 2.3.3.5, and 2.3.3.1), further constraining acetyl-CoA consumption via anaplerotic bypasses (Fig. 6).

Beyond metabolic reallocation, BTEX stress disrupted the biosynthesis of fundamental structural components. Proteins involved in gluconeogenesis, UDP-N-acetyl-α-D-glucosamine formation, and the synthesis of major cell wall constituents—peptidoglycan, teichoic acids, and lipopolysaccharides—showed declines (Fig. 6). Likewise, the expression of genes and proteins responsible for phospholipid biosynthesis, a key determinant of membrane integrity, was reduced. Collectively, these results indicate that BTEX exposure induces a redistribution of acetyl-CoA flux, favoring RBO-mediated carbon elongation and repressing oxidative metabolism and cell envelope biosynthesis.

### BTEX-induced regulatory strategies in CE

**Figure 7:**
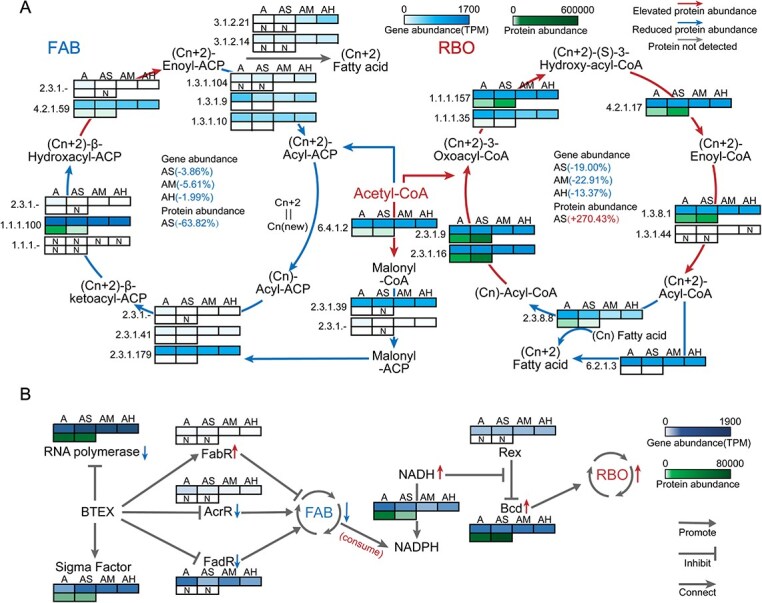
Gene and protein profiles in CE-related pathways. (A) Abundances of genes and proteins involved in the FAB and reverse RBO pathways. (B) Impact of BTEX exposure in the AS group on FAB and RBO pathway activity, illustrating that BTEX suppresses FAB (thereby conserving NADH) and alleviates Rex-mediated inhibition of Bcd, a key enzyme in the RBO pathway. Gene abundances are expressed in TPM, and protein abundances in LFQ intensities.

BTEX-induced metabolic reprogramming prompted a closer examination of the CE-associated FAB and RBO pathways. Multi-omics analyses revealed profound regulatory shifts within CE metabolic networks under BTEX exposure. FAB-associated proteins accounted for less than one-tenth of RBO abundance and declined further with BTEX, whereas RBO components, particularly in the AS group, were upregulated (Fig. 7A).

As protein abundance in prokaryotes is largely transcriptionally controlled, we next examined the dynamics of the transcriptional machinery (Fig. 7B). BTEX exposure increased the gene abundance of RNA polymerase and σ-factors, yet in the AS group, RNA polymerase proteins declined, whereas σ-factors remained stable. These trends suggest that BTEX represses global transcription, whereas maintaining selective transcriptional activity for CE-related genes.

Transcriptional regulators associated with FAB showed distinct responses to BTEX. Although corresponding protein data were unavailable, gene-level analyses revealed that the inhibitory regulator *fabR* [29] was upregulated in the AS group but downregulated in AM and AH, whereas the positive regulators *acrR* [30] and *fadR* [31] decreased across all treatments. These regulatory patterns are consistent with a targeted suppression of FAB, a process requiring greater ATP and NADPH input than RBO. In addition, the key RBO enzyme butyryl-CoA dehydrogenase (Bcd; EC 1.3.8.1) exhibited a pronounced increase in protein abundance, underscoring its pivotal role in BTEX-enhanced CE.

In parallel, the abundance of proton-translocating NAD(P) + transhydrogenase proteins declined markedly in the AS group, suggesting altered intracellular redox regulation. Consistent with this, intracellular assays on Day 12 revealed elevated NADH concentrations and NADH/NAD+ ratios in BTEX-exposed microcosms compared to the control (Fig. S7). Despite similar ethanol consumption across treatments, caproic acid accumulation was enhanced under BTEX exposure (Fig. 1D), indicating that elevated NADH availability may have favored RBO-mediated CE over FAB.

## Discussion

### BTEX promotes and integrates into soil CE

This study reveals that BTEX exposure reprograms soil microbial CE networks, coupling pollutant response with reductive carbon metabolism and driving shifts in microbial community organization. These findings extend the conventional understanding of pollutant–carbon interactions beyond the unidirectional paradigm of degradation and mineralization [32] toward a bidirectional metabolic coupling system that simultaneously facilitates pollutant detoxification and carbon retention.

At the metabolic level, low concentrations of BTEX markedly promoted the synthesis of MCFAs, particularly caproic acid, enhancing their production rate, yield, and persistence within the soil microcosm (Fig. 1D and E). The sustained accumulation of MCFAs indicates that microbial communities redirected reducing power toward reductive carbon conversion rather than oxidative mineralization, thereby improving carbon preservation efficiency under hydrocarbon stress. Such metabolic rerouting likely reflects an adaptive energy-conserving and redox-stabilizing response that helps maintain metabolic balance under hydrocarbon stress.

At the ecological level, BTEX exposure induced a functional reorganization of the microbial community, favoring syntrophic consortia that enhance CE efficiency. *Clostridium sensu stricto 12* [33] and *Rummelibacillus* [34] became dominant CE performers, replacing *Acinetobacter*—the leading taxon in the BTEX-free system—indicating the selective enrichment of CE-active anaerobes under aromatic stimulation (Fig. 2B). In contrast, *Petrimonas* proliferated only during the late stages of BTEX-free incubation, likely mediating secondary degradation of accumulated MCFAs [35] and thus contributing to reduced carbon retention (Fig. 2B).

**Figure 8:**
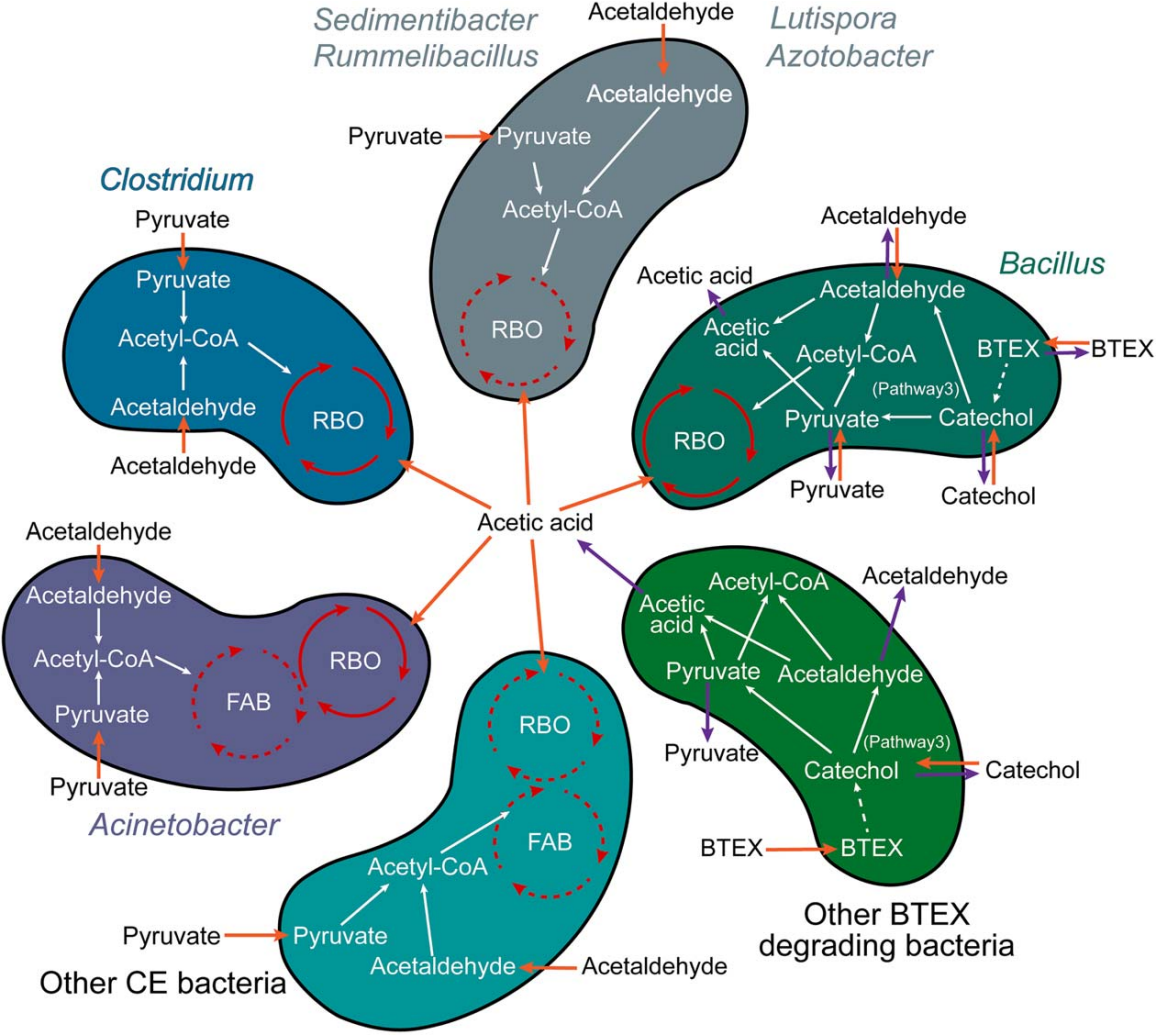
Association between CE and BTEX metabolism across bacterial genera. The heatmap illustrates temporal shifts in genus-level relative abundance. In the RBO and FAB pathways, solid lines: complete gene sets with ≤1 missing protein; dashed lines: complete genes but ≥2 missing proteins. For *Acinetobacter*, both gene and protein for a single FAB terminal-step enzyme were absent; this case is likewise represented by a dashed line.

The addition of BTEX compounds promotes metabolic cooperation between CE and BTEX-degrading microorganisms (Fig. 8). This interaction is supported by the coenrichment of CE- (Fig. 3) and BTEX-degrading (Fig. 4) pathways among dominant taxa at both the genus and protein levels. CE-related genus, including *Clostridium* and *Azotobacter*, can utilize transmembrane intermediates such as pyruvate and acetaldehyde derived from BTEX degraders, thereby establishing a substrate-based metabolic linkage between the two functional guilds. In this study, *Sedimentibacter* and *Lutispora* were found to possess genetic potential associated with CE metabolism (Fig. 3), indicating that these taxa may also function as elongators. Moreover, the genus *Bacillus* occupies a pivotal metabolic niche that bridges aromatic hydrocarbon degradation (Fig. 4) with the CE pathway (Fig. 3), facilitating redox coupling and metabolic symbiosis between degraders and elongators. Such a functional division of labor minimizes electron dissipation, enhances system-wide carbon conversion efficiency, and reveals a microbial mechanism that may contribute to pollutant-driven soil carbon stabilization.

### Multiple molecular mechanisms underlying BTEX-induced promotion of CE

Multi-omics analyses revealed that BTEX, which functions as a metabolic modulator rather than a mere carbon source or stressor, stimulates CE through a hierarchical mechanism, which involves accelerated substrate utilization, redirected acetyl-CoA allocation, and finely tuned regulation of redox and RBO pathways.

#### Substrate uptake and transformation

We observed that low and moderate BTEX concentrations exerted minimal effects on acetate transporter abundance, whereas high BTEX exposure substantially increased its expression (Fig. 5). Concurrently, BTEX exposure upregulated genes and proteins involved in flagellar biosynthesis and regulation. Given the central role of flagella in chemotaxis, substrate navigation, and environmental sensing [36], these results suggest that BTEX serves as a behavioral cue that elicits microbial responses to enhance physical access to diffusible carbon substrates. Beyond exogenous carbon utilization, BTEX also regulated endogenous carbon fixation pathways in a concentration-dependent manner. Under low BTEX stress (AS), the expression of genes and proteins related to the Wood–Ljungdahl pathway and other CO₂ fixation routes increased, indicating enhanced autotrophic carbon assimilation (Fig. 5). Within the ethanol catabolism module, BTEX remodeled carbon routing by enriching genes and proteins involved in ethanol-to-acetyl-CoA conversion and repressing the competing branch converting acetaldehyde to acetate (Fig. 5). These shifts collectively facilitated the generation and turnover of acetyl-CoA, the essential precursor for CE initiation.

#### Acetyl-CoA allocation

BTEX exposure fundamentally reprogrammed the utilization of acetyl-CoA, driving microbial metabolism toward CE rather than oxidative or biosynthetic pathways. Under low-to-moderate BTEX stress, the RBO pathway—the core driver of CE—was selectively enhanced, suggesting that reductive carbon flux was preferentially directed toward MCFA synthesis (Fig. 6). In contrast, oxidative pathways such as the TCA cycle and the glyoxylate shunt were suppressed, limiting acetyl-CoA oxidation. Simultaneously, BTEX exposure inhibited biosynthetic processes related to cell envelope formation (Fig. 6). Such inhibition of peptidoglycan, teichoic acid, and lipid metabolism may compromise cellular integrity and proliferation [37] but strengthens the allocation of metabolic energy toward reductive carbon retention.

#### CE pathway regulation

BTEX exposure triggered a system-wide reprogramming of CE-associated metabolic networks, characterized by coordinated control of energy and redox metabolism. Multi-omics evidence indicated that BTEX promoted CE not by expanding the genetic pool of CE-related genes but by enhancing the transcriptional and translational efficiency, producing higher functional protein yields from limited genetic resources (Fig. 7A). This prioritized transcriptional regulation reflects a microbial strategy of metabolic resource reallocation under chemical stress, wherein energy-intensive FAB is suppressed and the RBO system is reinforced to sustain reductive carbon flux. Compared with RBO, FAB demands greater ATP and NADPH consumption [38]. Under conditions where short-chain alcohols serve as primary substrates, their conversion to acetyl-CoA generates substantial NADH [39]. In this scenario, NADPH biosynthesis mainly depends on the NAD(P)+ transhydrogenase–mediated conversion of NADH rather than the pentose phosphate pathway. Thus, inhibiting FAB not only limits NADPH expenditure but also conserves NADH. In addition, the RBO key enzyme Bcd is transcriptionally regulated by Rex, a redox-sensitive regulator whose inhibitory activity weakens as NADH levels increase [40, 41]. By Day 12, the elevated NADH availability in the AS group alleviated Rex-mediated repression, thereby maintaining RBO-driven CE. In contrast, CE activity in the control (A) group stagnated and declined, as evidenced by the reduced accumulation of caproic acid (Fig. 1D).

### Threshold-dependent modulation of CE under BTEX stress

The effect of BTEX on CE exhibited a clear concentration dependence (Fig. 1D), reflecting a trade-off between adaptive metabolic activation and toxic inhibition under pollutant stress. At low BTEX levels, molecular and metabolic signatures of mild chemical stress were evident, including enhanced substrate uptake, redirected carbon flux toward acetyl-CoA (Fig. 5), and elevated reducing equivalents such as NADH (Fig. S7). This increased reductive capacity likely mitigated redox-sensitive inhibition (e.g. Rex-mediated regulation of *bcd*), thereby facilitating RBO and the elongation of short-chain intermediates into MCFAs (Fig. 7B).

Concurrently, even mild BTEX exposure led to decreased expression of key enzymes in the TCA cycle and glyoxylate shunt, along with suppressed pathways for membrane and cell wall biosynthesis (Fig. 6), indicating substantial metabolic stress and potential oxidative damage. Such disruptions likely explain the slowed and diminished MCFA synthesis observed at higher BTEX concentrations (Fig. 1D).

Collectively, these results demonstrate a biphasic, threshold-dependent pattern in pollutant–microbe interactions: low-level BTEX exposure induces adaptive metabolic reprogramming and enhances reductive carbon flux toward MCFAs, whereas excessive exposure surpasses microbial tolerance and destabilizes core metabolic networks. Within the limits of China’s environmental safety threshold (AS), low BTEX levels may stimulate CE and promote soil carbon storage in the form of MCFAs, providing a mechanistic reference for global soil pollution management.

### Future research prospects

This study provides mechanistic insights into BTEX-mediated regulation of the CE pathway in soil microbiomes, although inherent limitations exist. At the molecular scale, analyses were limited to gene- and protein-level data. Future investigations combining large-scale, multisample metagenomes with MAG-resolved frameworks for integrated metatranscriptomic and metaproteomic profiling [42] could enable higher-resolution reconstruction of metabolic interactions and regulatory hierarchies within key functional guilds. Such integrative approaches would substantially enhance the quantitative resolution and mechanistic interpretability of CE network analysis, thereby strengthening the link between pollutant stress and microbial carbon metabolism.

At the ecosystem scale, soil properties—including organic matter composition, redox conditions, and baseline microbial community structure—vary across environmental contexts. Extending these investigations across diverse soil types will clarify ecosystem-specific responses and potential variability in pollutant–microbiome interactions [43]. Moreover, the integration of *in situ* monitoring with isotope-based natural soil tracer methods will enable a more systematic and predictive understanding of how BTEX-induced microbial redox dynamics influence soil biogeochemical resilience, carbon flux regulation, and long-term carbon storage.

### Environmental and ecological impact

BTEX is ubiquitously present in soils globally. Regulatory thresholds for BTEX concentrations are typically established through comprehensive evaluations encompassing toxicological properties, ecological risks, human exposure potential, and broader environmental impacts [44, 45]. These standards increasingly extend beyond human health to address disruptions in ecosystem functions. Soil constitutes one of the largest terrestrial carbon reservoirs and plays a central role in carbon sequestration, storage, and exchange within the global carbon cycle [46]. Its stability is critical for maintaining regional and global carbon balance, particularly in the context of carbon-neutrality goals [47]. Here, integrative multi-omics analyses revealed a concentration-dependent regulatory effect of BTEX on microbial CE, spanning from taxonomic shifts to underlying molecular mechanisms. These findings underscore the bidirectional regulatory role of BTEX in soil carbon storage and provide mechanistic insights for establishing environmentally relevant BTEX thresholds in soil ecosystems.

As key byproducts of fossil fuel combustion—one of the major sources of anthropogenic carbon emissions—BTEX compounds enter soils via atmospheric deposition, precipitation, and adsorption onto soil particles [48]. Once deposited, they interact with soil microbial communities, forming a critical interface between anthropogenic carbon input and natural carbon sinks. Our findings delineate the complex ecological consequences of BTEX exposure on microbial carbon transformations and establish a cross-scale framework linking fossil fuel combustion, BTEX accumulation in soils, microbial functional responses, and resultant changes in carbon sequestration efficiency. This study provides new insights into the deep coupling between anthropogenic activities and biogeochemical carbon cycling.

## Supplementary Material

Supporting_Information_(Clean)_wraf254

Table_S6_wraf254

Table_S7_wraf254

## Data Availability

The amplicon sequencing and metagenomic datasets generated in this study have been deposited in the NCBI Sequence Read Archive under the accession numbers PRJNA1273664 and PRJNA1273842. The metaproteomics mass spectrometry proteomics data have been deposited to the ProteomeXchange Consortium (http://proteomecentral.proteomexchange.org) via the iProX partner repository with the dataset identifier PXD064805.
