# Supplementary material for: Aromatic pollutants rewire soil microbial carbon fixation via chain elongation
Source: ISME J. 2025 Nov 25;19(1):wraf254. doi: 10.1093/ismejo/wraf254 (PMC12694403; doi:10.1093/ismejo/wraf254)
Supplement: Supporting_Information_(Clean)_wraf254 [file supporting_information_(clean)_wraf254.docx]

**Supporting Information**

**Aromatic Pollutants Rewire Soil Microbial Carbon Fixation via Chain Elongation**

Qing-Lian Wu, Tian Lan, Lin Deng, Jing-Wen Jia, Wei-Tong Ren, Hua-Zhe Wang, Juan-Shan Du, Nan-Qi Ren, Wan-Qian Guo*

State Key Laboratory of Urban-rural Water Resource and Environment, Harbin Institute of Technology, Harbin 150090, China

***Corresponding author:** Wan-Qian Guo

**The following are included as supporting information for this paper:**

Number of pages: 26

Number of Text: 7

Number of Figures:9

Number of Tables: 5

**Text S1 Chemical analytical methods**

**(1) Detection methods for acids and alcohols**

Short-chain alcohol, SCFA and MCFA were determined using a gas chromatograph (GC) (7890, Agilent Technologies, USA) equipped with a flame ionization detector (FID) and an HP-INNOWax polyethylene glycol (PEG) chromatography column (Agilent 19095N-123) of 30 m × 0.53 mm × 1 μm.

**(2) BTEX concentration detection method**

The concentration of BTEX was determined using a GC-2010 plus gas chromatograph (GC) equipped with a flame ionization detector, HS-10 headspace sampler (Shimadzu, Japan), and an InterCap-WAX fused silica column (30 m × 0.25 mm; Shimadzu, Japan). Nitrogen (99.99%) was used as carrier gas (pressure 59.0 kPa). 1 g of homogenized liquid-soil sample or soil sample, 100 µl of internal standard solution (1% fluorobenzene), and 10 ml of saturated NaCl solution were added to a headspace sample bottle (20 ml) and the following procedure was performed:

**(a) Headspace sampler programs:**

Heating equilibrium temperature 85°C, heating equilibrium time 50 min, sampling needle temperature 100°C, transfer line temperature 110°C.

**(b) Gas chromatograph programs:**

40°C held for 6 min, increased to 110°C at a temperature increase rate of 5 °C/min, held for 1 min, and then increased to 200°C at a temperature increase rate of 6 °C/min, held for 3 min. Inlet temperature: 250°C; Interface temperature: 230°C; Carrier gas: helium; Injection mode: split injection, split ratio: 5: 1: Column flow rate: 1.0 mI/min.

**(c) Mass spectrometer programs:**

Scanning range: 35 amu~300 amu, at least 5 scans per peak; Ionization energy: 70 eV; Ion source temperature: 230°C; Quaternary rod temperature: 150°C; Scanning mode: Single Ion Monitoring (SIM). Before using SIM, the retention time needs to be obtained using full scan mode (SCAN).

**Text S2 16S rRNA Genes Amplicon Sequencing and Analysis**

Total microbial DNA was extracted from the original soil (day 0) and samples from groups A, AS, and AH collected on days 8, 16, and 24 using the FastPure Soil DNA Isolation Kit (magnetic bead-based; MJYH, Shanghai, China) following the manufacturer’s instructions. DNA quality and concentration were assessed using 1.0% agarose gel electrophoresis and a NanoDrop2000 spectrophotometer (Thermo Scientific, USA). Extracted DNA was stored at −80°C until further processing. The V3–V4 hypervariable region of the bacterial 16S rRNA gene was amplified using primer pairs 338F (5′-ACTCCTACGGGAGGCAGCAG-3′) and 806R (5′-GGACTACHVGGGTWTCTAAT-3′) on a T100 Thermal Cycler (Bio-Rad, USA). PCR reactions (20 μL) contained 4 μL 5× FastPfu buffer, 2 μL dNTPs (2.5 mM), 0.8 μL of each primer (5 μM), 0.4 μL FastPfu polymerase, 10 ng of template DNA, and nuclease-free water. The thermal profile included an initial denaturation at 95°C for 3 min, followed by 27 cycles of 95°C for 30 s, 55°C for 30 s, and 72°C for 45 s, with a final extension at 72°C for 10 min. Amplicons were purified from 2% agarose gels using a PCR Clean-Up Kit (YuHua, China) and quantified using Qubit 4.0 fluorometry (Thermo Fisher Scientific, USA). Equimolar pooled amplicons were sequenced using the NextSeq 2000 platform (Illumina, USA) with paired-end 2 × 250 bp reads, performed by Majorbio Bio-Pharm Technology Co., Ltd. (Shanghai, China).

Demultiplexed reads were quality-filtered using fastp v0.19.6 [1] and merged with FLASH v1.2.7. [2] Reads were truncated when the average quality score in a 50 bp sliding window fell below 20, and those shorter than 50 bp or containing ambiguous bases were discarded. Overlapping paired-end reads longer than 10 bp with ≤20% mismatches were merged; non-overlapping reads were removed. Sequences were assigned to samples based on exact barcode matches and ≤2 bp mismatches in primers. High-quality sequences were clustered into operational taxonomic units (OTUs) at 97% similarity using UPARSE v7.1 [3], and the most abundant sequence in each OTU was designated as the representative. To control for differences in sequencing depth, samples were rarefied to 20,000 sequences each, maintaining an average Good coverage greater than 99%. Taxonomic classification of representative OTU sequences was performed using the RDP Classifier version v2.2 [4] against the SILVA v138 database with a confidence threshold of 0.7. Raw sequencing data generated in this study have been deposited in the NCBI Sequence Read Archive (SRA) under the accession number PRJNA1273664.

**Text S3 Species diversity analysis**

Operational taxonomic units (OTUs) were clustered at 97% sequence similarity. Rarefaction curves and alpha diversity indices (including Sobs, ACE, Chao, Shannon, PD and coverage) were calculated using mothur (v.1.30.2 <https://mothur.org/wiki/calculators/>). Curve plotting was performed in R (v3.3.1).

Beta diversity was assessed by generating pairwise distance matrices with QIIME2 (v2020.2.0). Hierarchical clustering was visualized using dendrograms constructed in R. Principal coordinates analysis (PCoA) based on Bray–Curtis dissimilarities was conducted in R, with group differences tested by ANOSIM. The dilution curve and PCOA were performed using the R vegan package (v2.4.3).

**Text S4 Metagenomic Analysis**

Total genomic DNA was extracted from A, AS, AM, and AH using the FastPure Soil DNA Isolation Kit (magnetic bead-based; MJYH, Shanghai, China). DNA concentration and purity were assessed with a TBS-380 fluorometer and NanoDrop2000 spectrophotometer, and DNA integrity was confirmed via 1% agarose gel electrophoresis. For metagenomic library preparation, DNA was fragmented to an average size of ~350 bp using a Covaris M220 ultrasonicator (Gene Company Limited, China). Libraries were constructed using the NEXTFLEX Rapid DNA-Seq Kit (Bioo Scientific, USA), with Illumina-compatible adapters ligated to the fragments. Paired-end sequencing (2 × 150 bp) was performed on an NovaSeq 6000 platform (Illumina) using the S4 Reagent Kit v1.5 (300 cycles) at Majorbio Bio-Pharm Technology Co., Ltd. (Shanghai, China).

Adapter sequences were removed and low-quality reads (length <50 bp or quality score <20) were filtered out using fastp v0.23.0. High-quality reads were de novo assembled, and open reading frames (ORFs) were predicted from assembled contigs using Prodigal [5]. ORFs ≥100 bp were translated into amino acid sequences using EMBOSS 6.6.0 and NCBI's standard genetic code.

A non-redundant gene catalog was constructed using CD-HIT v4.6.1 [6] at 90% sequence identity and 90% alignment coverage. Clean reads were mapped back to the gene catalog using SOAPaligner v2.21 with a 95% identity threshold to estimate gene abundance. Gene abundances were normalized as TPM (Transcripts Per Million) to account for both gene length and sequencing depth. TPM was calculated as follows:$TPM=\frac{(reads mapped to a{gene}/{gene}length in kb)}{\sum(reads mapped to a{ll gene}/{gene}length in kb)}\times{10}^{6}$.

For taxonomic annotation, representative genes were aligned to the NCBI NR database using DIAMOND v0.8.35 [7] with an e-value cutoff of 1e^−5^. Functional annotations were assigned using DIAMOND against the eggNOG database for COG classification, and against the KEGG database for metabolic pathway analysis, both with an e-value cutoff of 1e^−5^. Raw sequencing data generated in this study have been deposited in the NCBI SRA under the accession number PRJNA1273842. Relevant sequencing metadata and gene annotation data are available in Tables S6 and S7.

**Text S5 Metaproteomic Analysis**

Proteins were extracted from frozen samples using BPP buffer and mechanical homogenization (3 × 40 s), followed by sequential phenol extraction and precipitation with ammonium acetate in methanol at –20°C. Pellets were washed with 90% acetone, redissolved in lysis buffer (8 M urea, 1% SDS, protease inhibitors), and protein concentrations were quantified using the BCA method (Thermo Scientific). For digestion, 100 μg of protein was reduced with 10 mM TCEP (37°C, 1 h), alkylated with 40 mM iodoacetamide (room temperature, 40 min, dark), centrifuged, resuspended in 100 mM TEAB, and digested with trypsin (1:50, w/w) overnight at 37°C. Peptides were desalted using HLB cartridges, dried, reconstituted in 0.1% TFA, and quantified via NanoDrop One. DIA-MS was performed on an Orbitrap Astral mass spectrometer (Thermo Scientific, USA) coupled with a Vanquish Neo LC system using a uPAC High Throughput column (75 μm × 5.5 cm) under an 8 min gradient. DIA data (MS1: 70–1050 m/z, MS2: 150–2000 m/z) were processed with Spectronaut v19 against a metagenomic protein database. Search parameters included: trypsin/P, max two missed cleavages, peptide length 7–52, fixed modification (carbamidomethyl-C), variable modifications (oxidation-M, N-terminal acetylation), FDR ≤ 1%, and peptide confidence ≥ 99%. Quantification used the MaxLFQ algorithm. Differentially expressed proteins (DEPs) were identified (p < 0.05, fold change >1.2 or <0.83), and functional enrichment analyses were conducted using GO and KEGG databases. Protein-protein interaction networks were constructed using STRING v11.5. Protein abundance is reported as LFQ intensity (Label-Free Quantification intensity) (MaxQuant output), which is MS1-derived normalized intensity value expressed in arbitrary units (a.u.). The metaproteomics mass spectrometry proteomics data have been deposited to the ProteomeXchange Consortium (http://proteomecentral.proteomexchange.org) via the iProX [8] partner repository with the dataset identifier PXD064805.

**Text S6 Widespread, Concentration-Dependent Effects of BTEX on Soil CE**

This large-scale assessment more systematically delineated the effects of varying BTEX concentrations on soil CE activity (Fig. S8). For the maximum accumulation of medium-chain fatty acids (MCFAs; Fig. S9), Shapiro–Wilk tests indicated that Groups A (W = 0.857, *P* = 0.088), AS (W = 0.871, *P* = 0.125), and AM (W = 0.935, *P* = 0.529) conformed to normal distributions, whereas Group AH (W = 0.825, *P* = 0.039) did not. Consequently, independent-sample t-tests were applied to normally distributed groups. Group AS exhibited significantly higher MCFAs accumulation than Group A (t = –2.13, df = 16, *P* = 0.049) under homogeneity of variance (Levene’s test, *P* = 0.087), suggesting that mild BTEX exposure significantly promoted caproic acid synthesis despite intrinsic variability in soil composition and microbial structure. In contrast, no significant difference was observed between Groups A and AM (t = 0.19, df = 16, *P* = 0.852; Levene’s test, *P* = 0.134). For the non-normally distributed Group AH, the Mann–Whitney U test (U = 34.0, *P* = 0.605) indicated that high BTEX concentrations suppressed CE activity but did not significantly reduce total MCFA production, warranting further validation in larger datasets.

**Text S7 BTEX Exposure Reshapes Soil Microbial Diversity and Phylum-Level Succession Dynamics**

The dominant phyla across all groups (Fig. 2A) and time points were *Firmicutes*, *Proteobacteria*, *Actinobacteriota*, *Acidobacteriota*, and *Bacteroidota*. Despite their shared dominance, these phyla exhibited divergent temporal patterns across treatments. *Firmicutes*, a phylum frequently associated with CE processes [9, 10], showed notable fluctuations in groups A and AM. In group AS, its relative abundance declined moderately over time, likely due to the depletion of electron donors such as ethanol and acetate, although it remained the dominant phylum at 59.67%. In contrast, group AH displayed a marked increase in Firmicutes from 15.90% at the initial stage to 72.99% by day 24. A distinct inflection occurred on day 16, with abundance rising to 31.42%, coinciding with the onset of caproic acid accumulation (Fig. 1C). This delayed enrichment suggests the emergence of CE activity in AH during the later phase. Bacteroidota displayed a different pattern. In group A, this phylum increased rapidly, with relative abundance increasing from 0.03% on day 8 and 0.09% on day 16 to 23.65% by day 24. In contrast, *Bacteroidota* remained rare in groups AS, AM, and AH, reaching only 0.31%, 0.12%, and 0.18%, respectively, by day 24. Previous studies have implicated *Bacteroidota* in the degradation of complex biomacromolecules [11, 12]. Therefore, their proliferation in group A may reflect the degradation of MCFAs during the late stages of incubation. This inference aligns with the concurrent decline in caproic acid levels observed in group A (Fig. 1C), suggesting that BTEX exposure may contribute to MCFAs stabilization by suppressing the proliferation and metabolic activity of *Bacteroidota*.


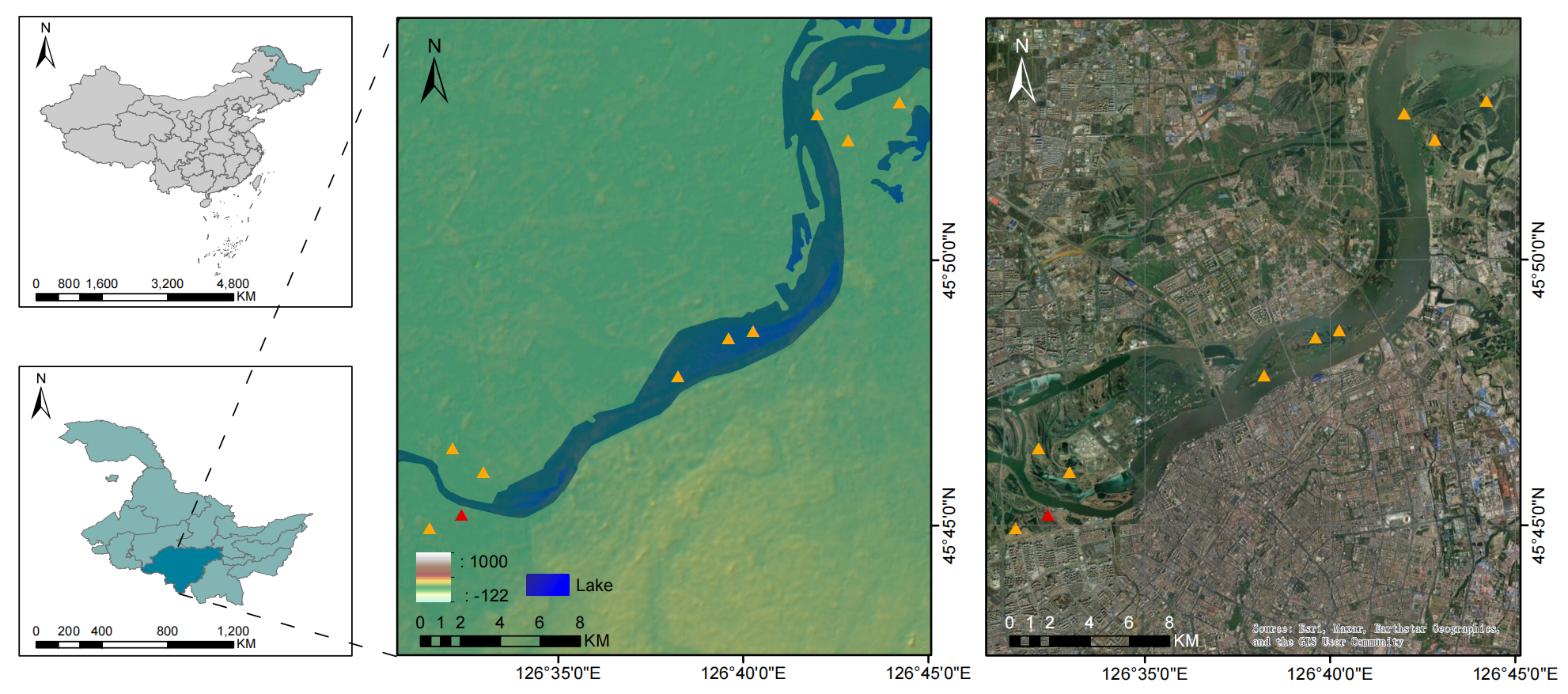


Figure S1 Schematic representation of sampling locations. Red denotes sites sampled in September 2024, and orange denotes sites sampled in September 2025.


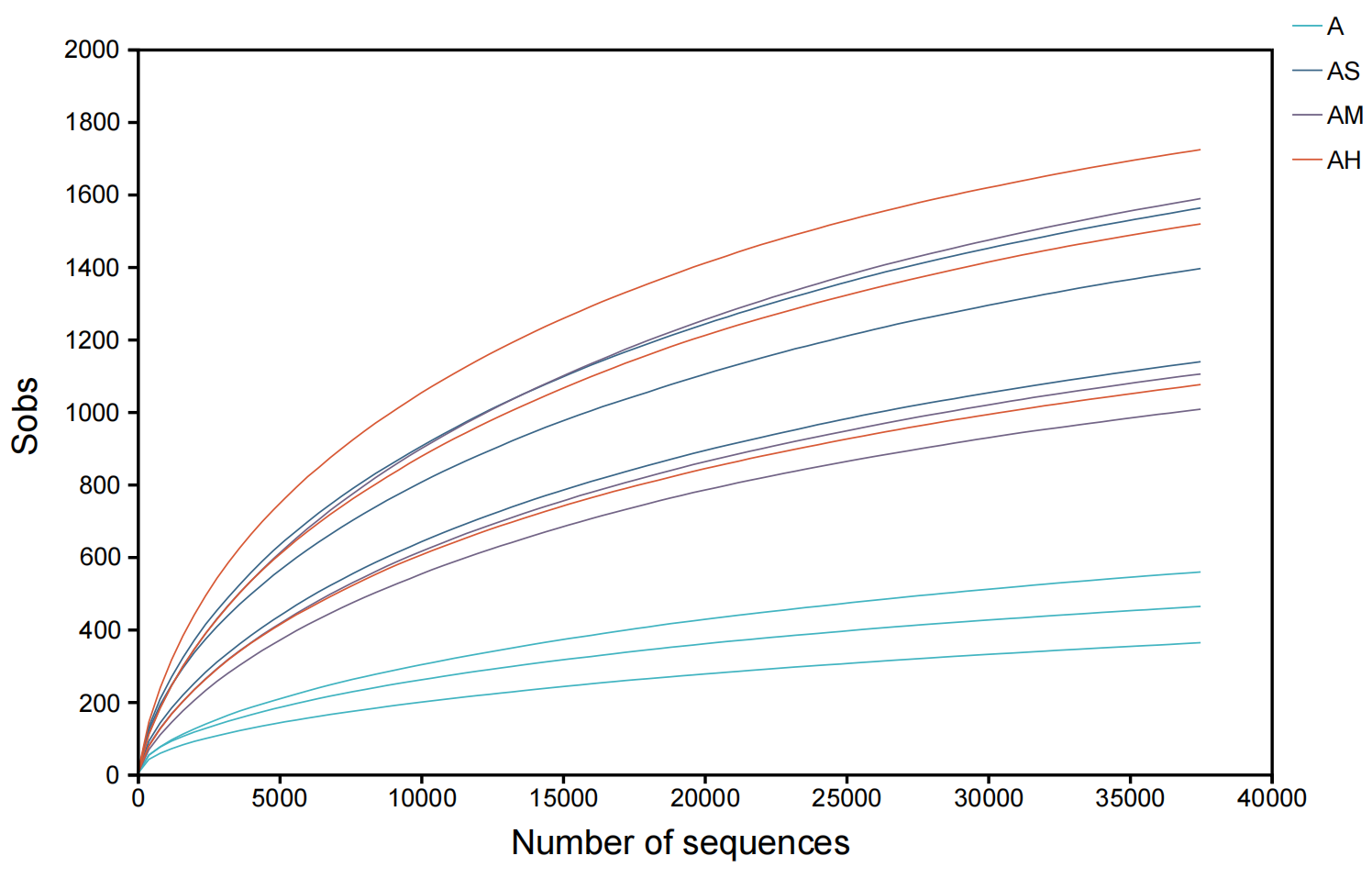


Figure S2 Rarefaction curves based on the observed species (Sobs) index across all samples, illustrating sequencing depth and species richness saturation.


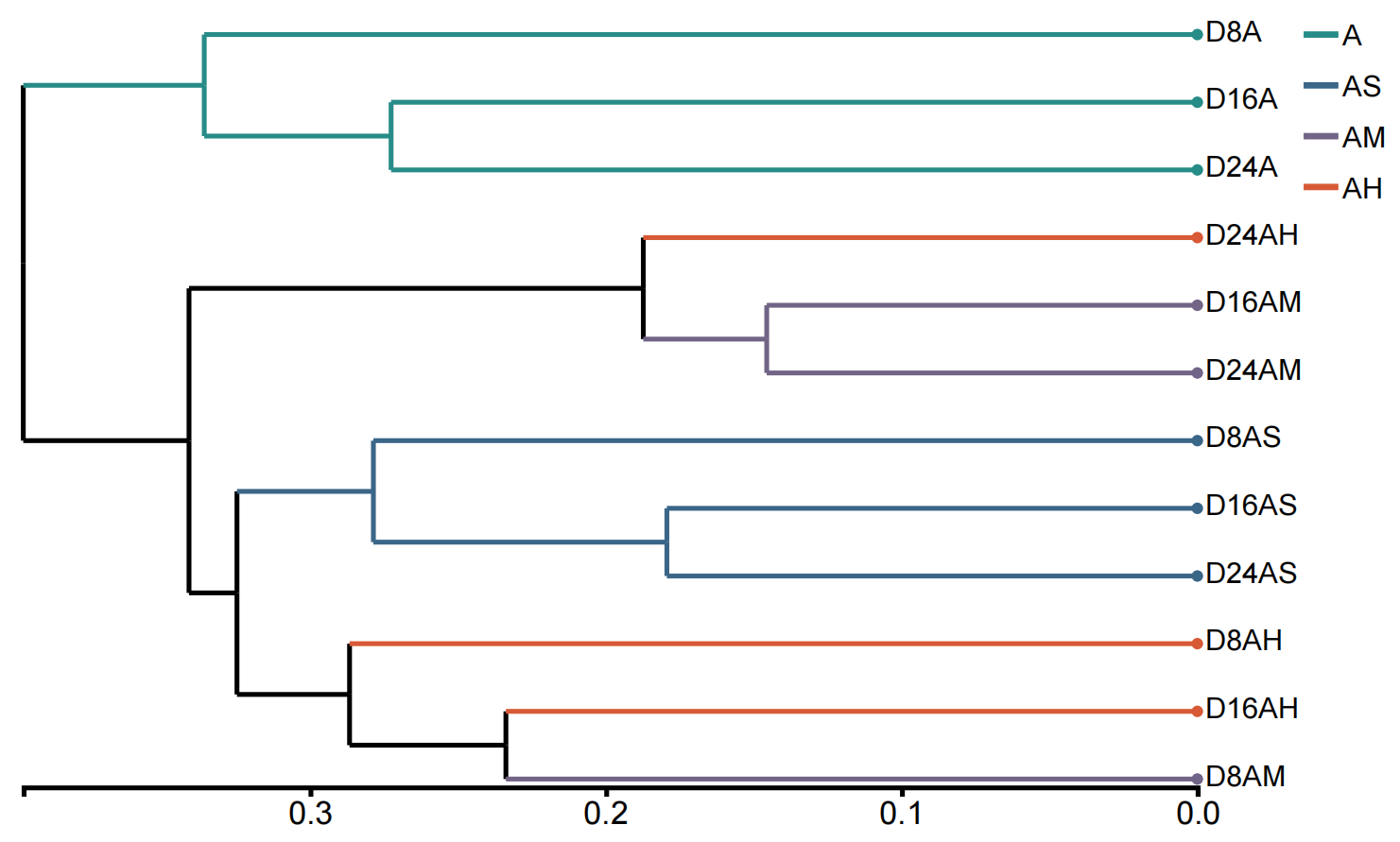


Figure S3 Hierarchical clustering tree based on OTU-level Bray–Curtis dissimilarities among the A, AS, AM, and AH samples across three time points.


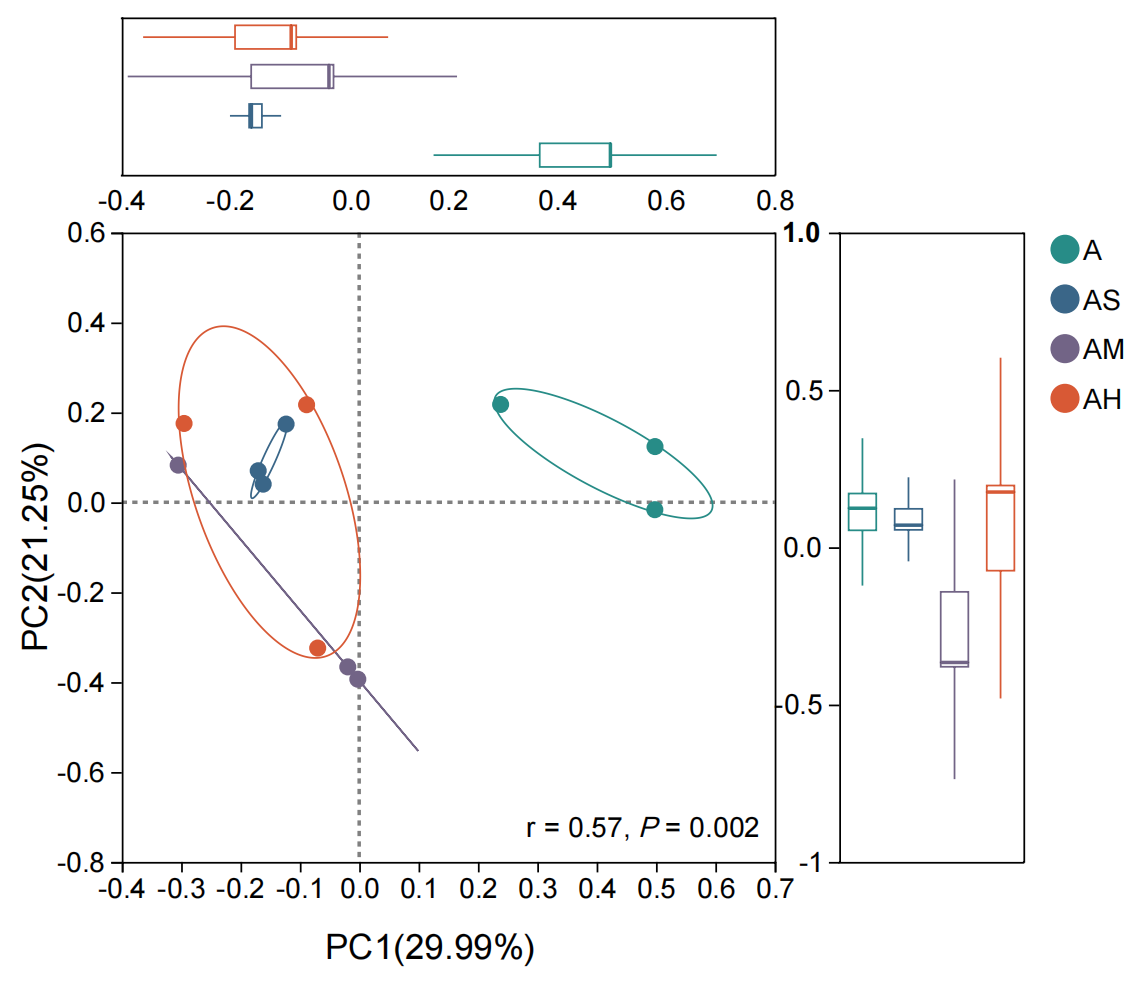


Figure S4 PCoA based on Bray–Curtis dissimilarity revealed clear segregation of microbial communities, with the non-BTEX group (A) differing significantly from BTEX-treated groups (AS, AM, AH), as supported by ANOSIM (r = 0.5679, *P* = 0.0020).


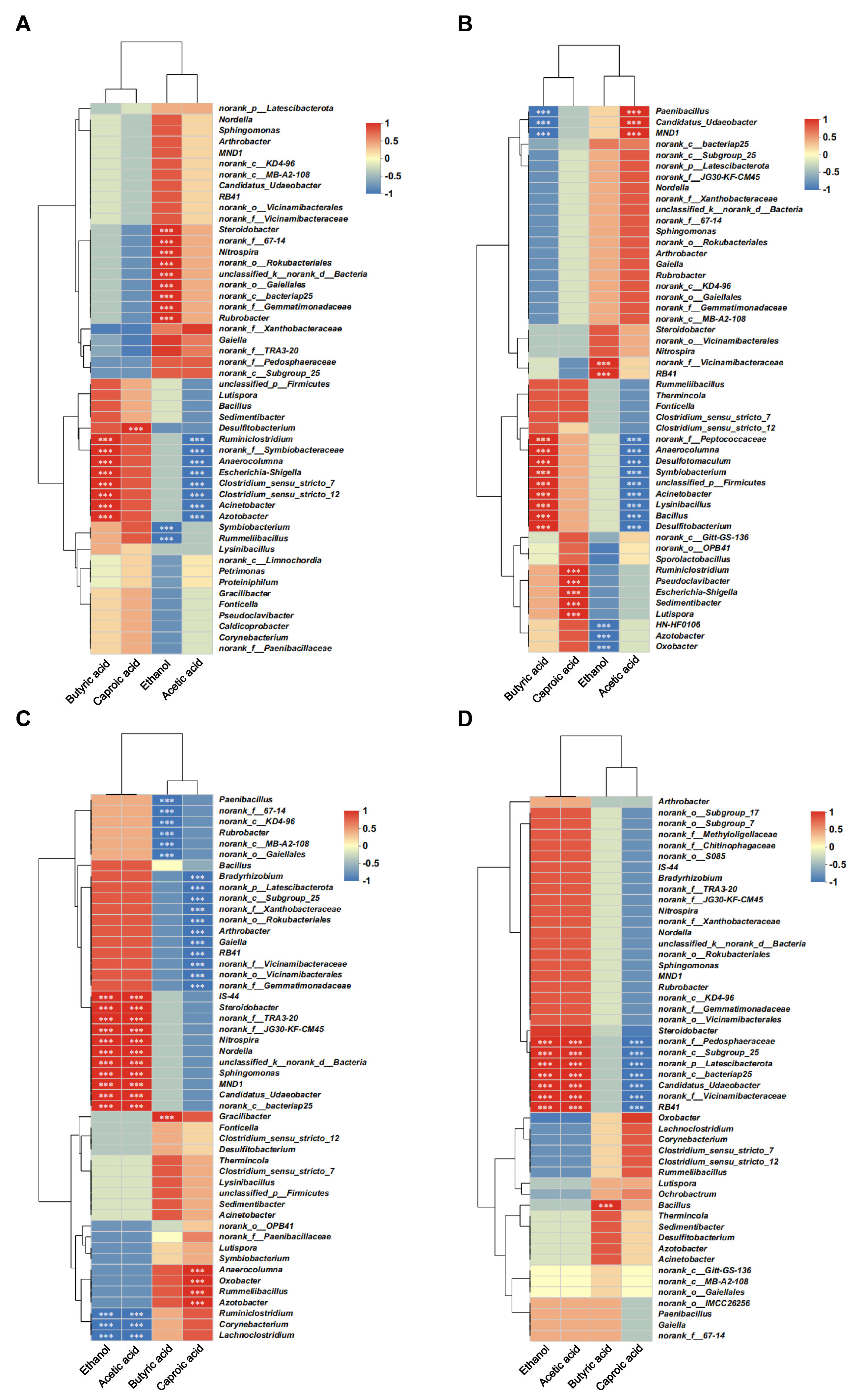


Figure S5 Spearman correlation analysis between the concentrations of ethanol, acetic acid, butyric acid, and caproic acid and the bacterial community composition at each stage under different BTEX treatments. Panels represent individual treatments: (A) A; (B) AS; (C) AM; (D) AH.


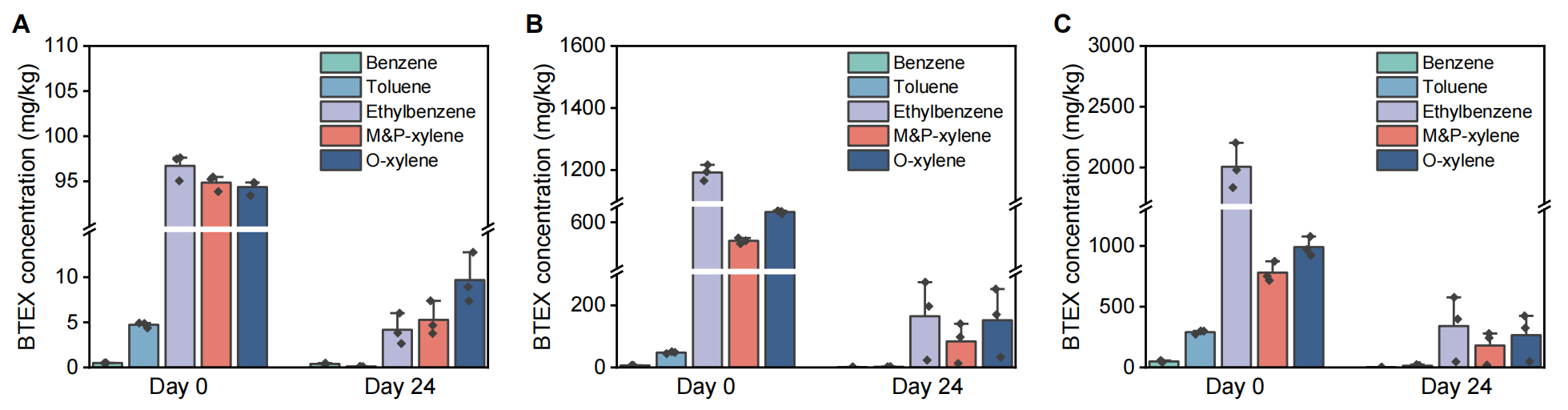


Figure S6 Concentrations of individual BTEX compounds at the beginning and end of the experiment in treatments AS (A), AM (B), and AH (C), respectively.


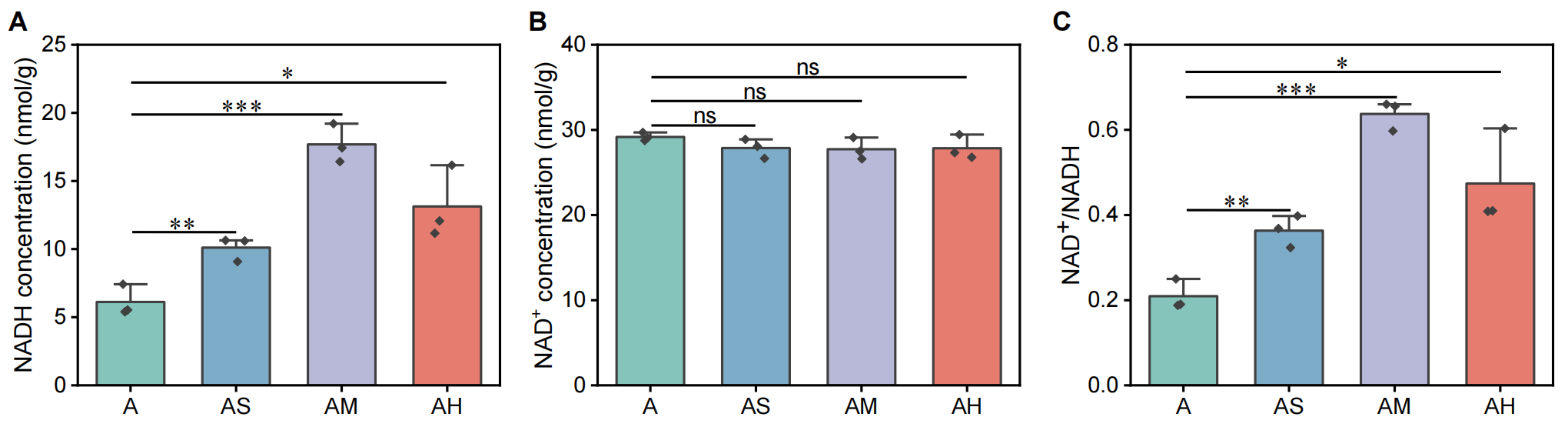


Figure S7 Quantification of intracellular NADH and NAD⁺ levels, and their molar ratio (NADH/NAD⁺) in treatments A, AS, AM, and AH on day 12. (a) NADH concentration; (b) NAD⁺ concentration; (c) NADH/NAD⁺ ratio. (* indicates *P* < 0.05, ** indicates *P* < 0.01, *** indicates *P* < 0.005)


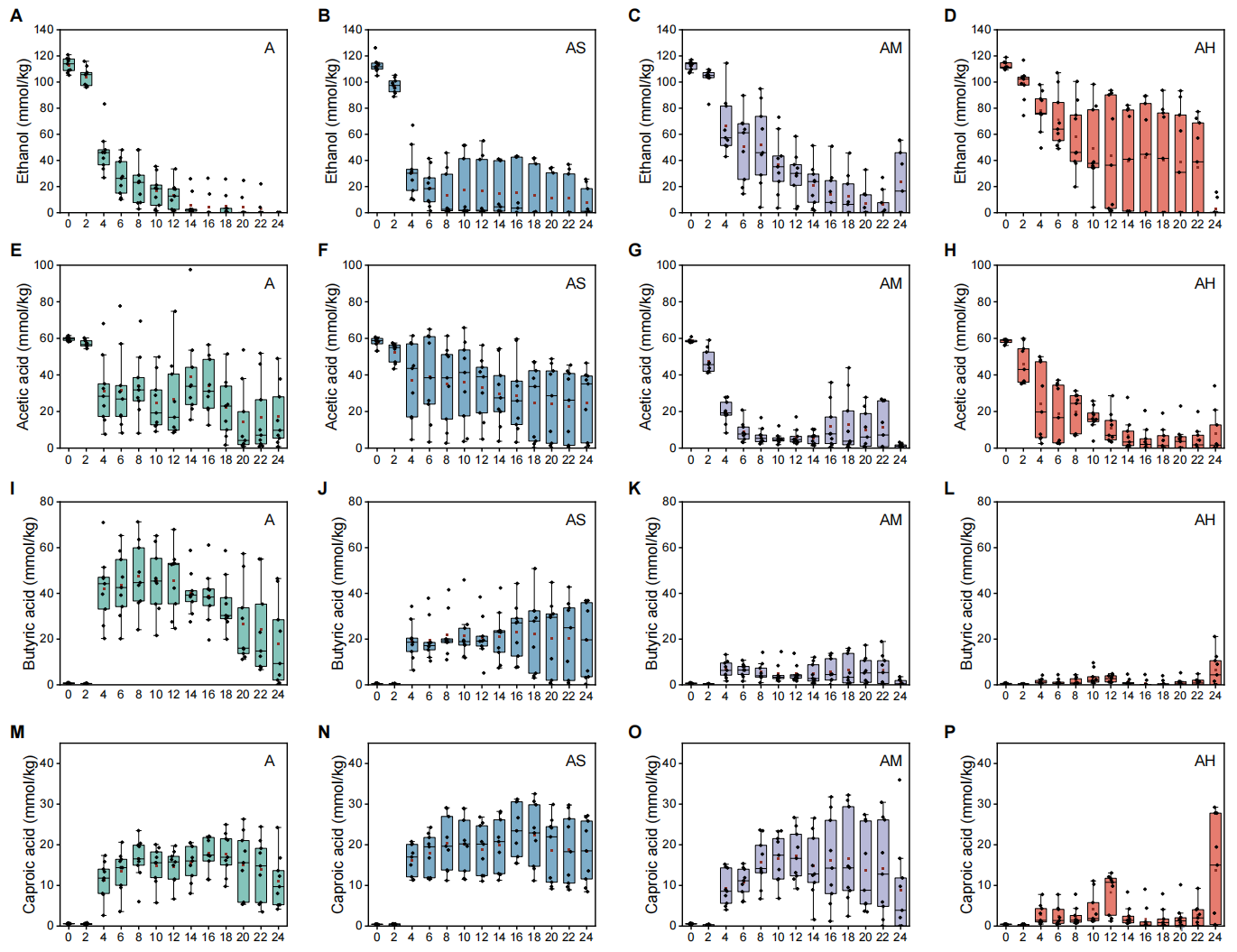


Figure S8 Temporal variations in substrate and product concentrations across Group A (green), Group AS (blue), Group AM (purple), and Group AH (red). Panels (A–D) depict ethanol dynamics, (E–H) acetic acid, (I–L) butyric acid, and (M–P) caproic acid, respectively.


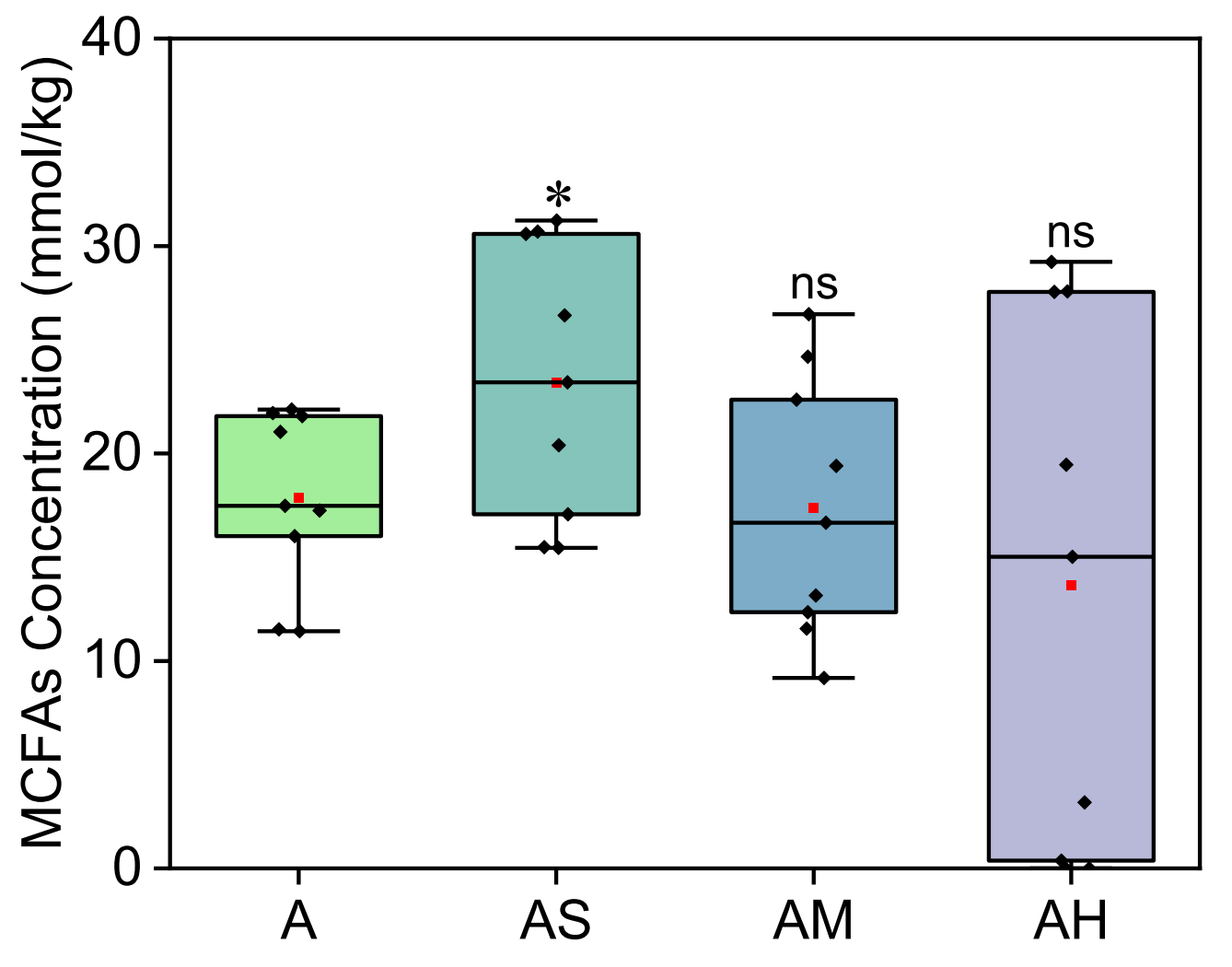


Figure S9 Maximum accumulation of MCFAs in Groups A, AS, AM, and AH. MCFAs production in Group AS was significantly higher than that in Group A (* indicates *P* < 0.05).

**Table S1** BTEX concentrations corresponding to different levels of contamination

| mg/kg | slight pollution | Moderate pollution | | Heavy pollution |
| --- | --- | --- | --- | --- |
| Benzene | 0.5 | 7 | 50 | |
| Toluene | 5 | 50 | 300 | |
| Ethylbenzene | 100 | 1200 | 2000 | |
| M-Xylene | 50 | 270 | 400 | |
| P-Xylene | 50 | 270 | 400 | |
| O-Xylene | 100 | 640 | 1000 | |

**Table S2** Medium composition

| Component | Concentration |
| --- | --- |
| NH_4_Cl | 2.04 g/L |
| MgCl_2_ 7H_2_O | 0.2 g/L |
| KH_2_PO_4_ | 0.23 g/L |
| K_2_HPO_4_ | 0.31 g/L |
| NaCl | 0.8 g/L |
| CaCl_2_ 2H_2_O | 0.2 g/L |
| L-Cysteine | 0.25 g/L |
| Sodium 2-bromoethanesulfonate | 5 g/L |

**Table S3** Layout of Experimental Treatments

|  | A | AS | AM | AH |
| --- | --- | --- | --- | --- |
| Pollution level | - | slight | moderate | heavy |
| Ethanol (mmol/kg) | 125.29 (±0.74) | 125.74 (±2.36) | 124.43 (±2.95) | 126.50 (±1.59) |
| Acetic acid (mmol/kg) | 57.69 (±0.98) | 56.81 (±0.23) | 57.66 (±0.63) | 57.57 (±0.84) |
| Benzene (mg/kg) | 0 | 0.49 (±0.02) | 6.59 (±0.45) | 49.44 (±7.58) |
| Toluene (mg/kg) | 0 | 4.72 (±0.30) | 47.72 (±3.23) | 290.58 (±15.15) |
| Ethylbenzene (mg/kg) | 0 | 96.71 (±1.44) | 1192.22 (±25.73) | 2005.47 (±185.67) |
| M&P -xylene (mg/kg) | 0 | 94.87 (±0.87) | 539.99 (±9.20) | 780.23 (±83.23) |
| O-xylene (mg/kg) | 0 | 94.37 (±0.81) | 632.86 (±5.27) | 990.64 (±79.50) |

As m-xylene and p-xylene could not be adequately separated by GC-MS, their concentrations were combined and reported as a single measurement.

|  | mmol/kg | mg/kg |
| --- | --- | --- |
| Ethanol | 1 | 46.0684 |
| Acetic acid | 1 | 60.0520 |
| Butyric acid | 1 | 88.1051 |
| Caproic acid | 1 | 116.1583 |
| Benzene | 0.0128 | 1 |
| Toluene | 0.0108 | 1 |
| Ethylbenzene | 0.0094 | 1 |
| M-xylene | 0.0094 | 1 |
| P-xylene | 0.0094 | 1 |
| O-xylene | 0.0094 | 1 |

**Table S4** Conversion of quantitative units

**Table S5** Alpha diversity index

| Sample | Sobs | Ace | Chao | Shannon | Pd | Coverage |
| --- | --- | --- | --- | --- | --- | --- |
| D8A | 558 | 821.83 | 781.20 | 2.87 | 64.80 | 0.9942 |
| D8AS | 1138 | 1511.67 | 1415.17 | 3.80 | 109.65 | 0.9900 |
| D8AM | 1588 | 2054.99 | 1940.99 | 4.01 | 148.71 | 0.9867 |
| D8AH | 1518 | 1919.64 | 1814.68 | 3.33 | 141.93 | 0.9880 |
| D16A | 363 | 559.13 | 521.40 | 2.59 | 49.52 | 0.9962 |
| D16AS | 1562 | 2024.79 | 1942.33 | 4.63 | 138.90 | 0.9869 |
| D16AM | 1007 | 1350.41 | 1281.40 | 2.89 | 101.73 | 0.9908 |
| D16AH | 1723 | 2075.88 | 1974.54 | 4.30 | 151.48 | 0.9884 |
| D24A | 463 | 653.048 | 647.17 | 3.13 | 56.45 | 0.9955 |
| D24AS | 1395 | 1830.04 | 1741.63 | 4.33 | 133.94 | 0.9881 |
| D24AM | 1104 | 1492.11 | 1400.89 | 3.30 | 106.55 | 0.9899 |
| D24AH | 1075 | 1431.58 | 1359.34 | 3.14 | 103.36 | 0.9904 |

D for days

**References**

1. Chen S, Zhou Y, Chen Y, Gu J. Fastp: An ultra-fast all-in-one fastq preprocessor. *Bioinformatics*. 2018;**34**:i884-i90 <https://doi.org/10.1093/bioinformatics/bty560>

2. Magoč T, Salzberg SL. Flash: Fast length adjustment of short reads to improve genome assemblies. *Bioinformatics*. 2011;**27**:2957-63 <https://doi.org/10.1093/bioinformatics/btr507>

3. Edgar RC. Uparse: Highly accurate otu sequences from microbial amplicon reads. *Nat Methods*. 2013;**10**:996-98 <https://doi.org/10.1038/nmeth.2604>

4. Wang Q, Garrity GM, Tiedje JM, Cole JR. Naive bayesian classifier for rapid assignment of rrna sequences into the new bacterial taxonomy. *Appl Environ Microbiol*. 2007;**73**:5261-7 <https://doi.org/10.1128/aem.00062-07>

5. Hyatt D, Chen G-L, LoCascio PF *et al.* Prodigal: Prokaryotic gene recognition and translation initiation site identification. *BMC Bioinformatics*. 2010;**11**:119 <https://doi.org/10.1186/1471-2105-11-119>

6. Fu L, Niu B, Zhu Z *et al.* Cd-hit: Accelerated for clustering the next-generation sequencing data. *Bioinformatics*. 2012;**28**:3150-2 <https://doi.org/10.1093/bioinformatics/bts565>

7. Buchfink B, Xie C, Huson DH. Fast and sensitive protein alignment using diamond. *Nat Methods*. 2015;**12**:59-60 <https://doi.org/10.1038/nmeth.3176>

8. Chen T, Ma J, Liu Y *et al.* Iprox in 2021: Connecting proteomics data sharing with big data. *Nucleic Acids Res*. 2022;**50**:D1522-d27 <https://doi.org/10.1093/nar/gkab1081>

9. Mendoza MD, Vaca L, Erazo P, Villa P. Perspectives on carboxylates generation from ecuadorian agro-wastes. *Bioresour Technol*. 2024;**407**:131080 <https://doi.org/10.1016/j.biortech.2024.131080>

10. Huo W, Ye R, Hu T, Lu W. Co2 uptake in ethanol-driven chain elongation system: Microbial metabolic mechanisms. *Water Res*. 2023;**247**:120810 <https://doi.org/https://doi.org/10.1016/j.watres.2023.120810>

11. Muhammad N, Avila F, Kim SG. Comparative genome analysis of the genus marivirga and proposal of two novel marine species: Marivirga arenosa sp. Nov., and marivirga salinae sp. Nov. *BMC Microbiol*. 2024;**24**:245 <https://doi.org/10.1186/s12866-024-03393-3>

12. Fernández-Gómez B, Richter M, Schüler M *et al.* Ecology of marine bacteroidetes: A comparative genomics approach. *ISME J*. 2013;**7**:1026-37 <https://doi.org/10.1038/ismej.2012.169>
